# Inter-Organelle NAD Metabolism Underpinning Light Responsive NADP Dynamics in Plants

**DOI:** 10.3389/fpls.2019.00960

**Published:** 2019-07-26

**Authors:** Shin-nosuke Hashida, Maki Kawai-Yamada

**Affiliations:** ^1^Environmental Science Research Laboratory, Central Research Institute of Electric Power Industry, Abiko, Japan; ^2^Graduate School of Science and Engineering, Saitama University, Saitama, Japan

**Keywords:** NAD^+^ supply, NAD^+^ synthesis, NADP^+^ synthesis, light response, linear electron transfer, photosynthesis, chloroplasts

## Abstract

Upon illumination, photosystem I in chloroplasts catalyzes light-driven electron transport from plastocyanin to ferredoxin, followed by the reduction of NADP^+^ to NADPH by ferredoxin:NADP^+^ reductase for CO_2_ fixation. At the beginning of photosynthesis, NADP^+^ supply control is dominated by *de novo* NADP^+^ synthesis rather than being recycled from the Calvin cycle. Importantly, ferredoxin distributes electrons to NADP^+^ as well as to thioredoxins for light-dependent regulatory mechanisms, to cyclic electron flow for more adenosine triphosphate (ATP) production, and to several metabolites for reductive reactions. We previously demonstrated that the NADP^+^ synthesis activity and the amount of the NADP pool size, namely the sum of NADP^+^ and NADPH, varies depending on the light conditions and the ferredoxin-thioredoxin system. In addition, the regulatory mechanism of cytoplasmic NAD^+^ supply is also involved in the chloroplastic NADP^+^ supply control because NAD^+^ is an essential precursor for NADP^+^ synthesis. In this mini-review, we summarize the most recent advances on our understanding of the regulatory mechanisms of NADP^+^ production, focusing on the interactions, crosstalk, and co-regulation between chloroplasts and the cytoplasm at the level of NAD^+^ metabolism and molecular transport.

## Introduction

Nicotinamide adenine dinucleotide (NAD) and its phosphorylated form (NADP) are essential electron acceptors/donors in a broad range of cellular redox processes (Pollak et al., 2007). Interestingly, these two cofactors have rather distinct biological roles. NAD is mainly used in catabolic processes to produce cellular energy as an oxidant (Geigenberger, 2003), whereas NADP is often involved in anabolic processes to produce photosynthates, fatty acids, and carbon skeletons to support plant growth as a reductant (Kramer et al., 2004; Noctor et al., 2006). The major NADPH-generating source in darkness is the oxidative pentose phosphate pathway (OPPP) coupled to central carbon metabolism in chloroplasts (Kruger and von Schaewen, 2003). Redox regulation of OPPP enzymes in chloroplasts relies on thioredoxin (Trx) and NADPH-dependent Trx reductase C (NTRC) (Perez-Ruiz et al., 2017), thereby balancing the redox status for protection against oxidative damage (Perez-Ruiz et al., 2006).

**Figure 1:**
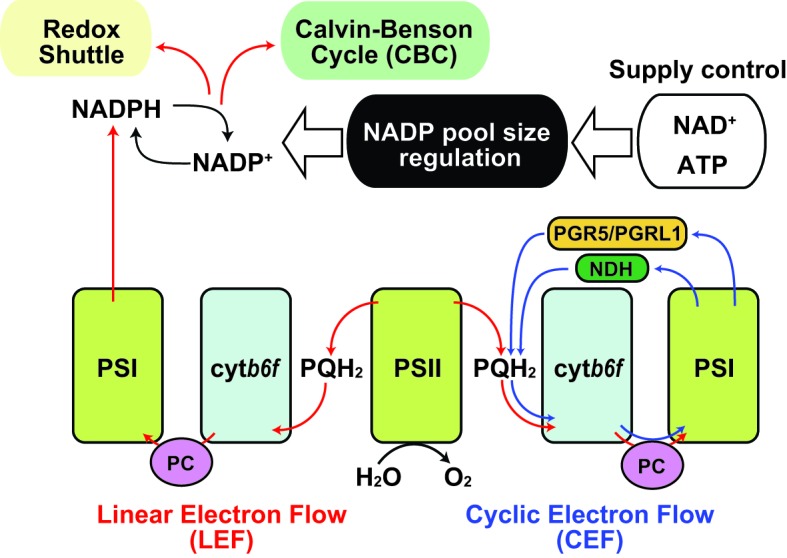
Regulation of NADP pool size in photosynthesis. Schematic representation of the relationship between NADP pool size regulation and photosynthetic electron flow was shown. Red arrow indicates linear electron flow (LEF), and blue arrow indicates cyclic electron flow. Black arrow indicates molecular conversion. PSI, photosystem I; PSII, photosystem II; cyt*b_6_f*, cytochrome *b_6_f* complex; PQH_2_, plastoquinol; PC, plastocyanin; PGR5, proton gradient regulation 5; PGRL1, PGR5-like 1; NDH, chloroplast NAD(P)H dehydrogenase-like complex.

Under sunlight, photosynthetic electron transfer chains (PETC) are the primary source of NADPH. Plants use sunlight as a primary energy source for photosynthesis in chloroplasts (Ort and Yocum, 1996). During this process, light drives electron transfer reactions by which protons are transferred from the stroma into the thylakoid lumen generating proton motive force (*pmf*) that is used for ATP synthesis (Kanazawa et al., 2017). Most *pmf* appears to be generated though linear electron flow (LEF) in which electrons released from water in photosystem II (PSII) are eventually transferred to NADP^+^ through photosystem I (PSI; Figure 1, red line; Joliot and Joliot, 2005). Thus, photosynthesis provides NADPH as reducing power for the Calvin-Benson cycle (CBC) to assimilate carbon dioxide. After use of the reducing power in CBC, NADP^+^ is again recycled back as an electron acceptor in PSI. However, under stress conditions that weaken CBC enzymatic activity, a declining rate of NADPH usage and NADP recycling occurs and LEF can be overloaded resulting in generation of reactive oxygen species from the photosystems (Hajiboland, 2014; Foyer, 2018). Hence, a number of studies have elucidated the protective mechanisms, including antioxidant production, antenna size regulation, and alternative electron flow, as regulatory mechanisms of photosynthesis under natural environments (Demmig-Adams and Adams, 1992; Takahashi and Badger, 2011; Pinnola and Bassi, 2018). For example, the “malate valve” is a representative redox shuttle system of the balancing redox state in chloroplasts and exclusively exports the excess reducing power of NADPH in chloroplasts to NAD^+^ in cytosol (Selinski and Scheibe, 2019), by which NADP^+^ could be replenished to PSI instead of CBC. Thus, NADP^+^ re-supply is crucial for the redox balancing system in chloroplasts. Here, a simple question arises: why is NADP^+^ additionally provided for LEF *via de novo* NADP^+^ synthesis in chloroplasts when or before it starves? To answer this, we need to understand the uncharacterized regulatory mechanisms of NADP^+^ synthesis in chloroplasts.

NAD^+^ is the only promising candidate to be the precursor for NADP^+^ synthesis and is converted to NADP^+^ by ATP-dependent NAD kinase (NADK) (McGuinness and Butler, 1985). Although NAD is exclusively produced in the cytosol (Hashida et al., 2009), NADP production is executed at *on demand sites* by various isoforms of NADK. For example in *Arabidopsis*, NADK1 is located in the cytosol, whereas NADK2 and NADK3 are targeted to chloroplasts and peroxisomes, respectively (Waller et al., 2009). Moreover, NADK1 and NADK2 use NAD^+^ as the preferred substrate, whereas NADK3 demonstrates a strong preference for NADH (Berrin et al., 2005; Chai et al., 2005, 2006). For decades, the Ca^2+^/Calmodulin (CaM)-dependence of NADK activity has been recognized (Muto and Miyachi, 1977; Anderson et al., 1980; Karita et al., 2004) and NADK2 was reported capable of binding to CaM *in vitro* (Turner et al., 2004). However, no candidate CaM and no response to Ca^2+^ in NADK2 activity have been reported elsewhere (Dell’Aglio et al., 2016). Instead of NADK2, it was recently reported that *Arabidopsis* P-loop ATPase has CaM-dependent NADK activity (Dell’Aglio et al., 2019). Rather than Ca^2+^, the light signal appears to control chloroplastic NADK2 activity and NADP^+^ production according to the current knowledge of NADP response to light conditions (Thormahlen et al., 2017). The goal of this mini-review is to highlight the importance of regulating NAD^+^ supply for chloroplastic NADP^+^ synthesis that is specific to photosynthetic organisms.

## Dynamics of NADP Pool Size in Response to Light Conditions

**Figure 2:**
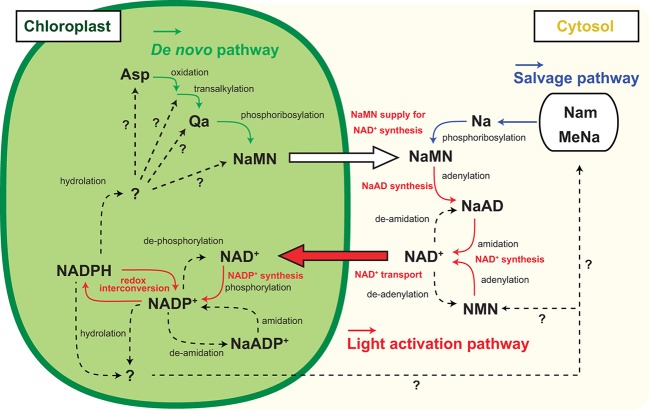
Inter-organelle nicotinamide adenine dinucleotide (NAD) metabolism associated with chloroplastic NADP dynamics. Schematic representation of NADP^+^ biosynthesis and NAD^+^ biosynthesis and part of their metabolism and inter-organelle transport. Solid arrow indicates the identified molecular conversion, and dashed arrow indicates unidentified molecular conversion. Green line highlights the *de novo* pathway, and blue line highlights the salvage pathway of NAD^+^ biosynthesis. Red line highlights the light activation pathway. Abbreviations are as shown in the text except for Nam, MeNa, and NMN indicating nicotinamide, methyl nicotinate, and nicotinamide mononucleotide, respectively.

According to the rationale of photosynthesis, light drives NADP^+^ reduction, meaning NADPH generation. Most typical illustrations concerning photosynthesis demonstrate the qualitative interpretation of the photochemical process but do not give any quantitative interpretations about NADP (Figure 1). Under steady-state exposure conditions, the balance between the generation and utilization of reducing power is equilibrated by complicated interactions between LEF, CBC, redox shuttle, and cyclic electron flow (CEF), which exclusively generate *pmf* contributing to ATP production (Munekage et al., 2004; Alric and Johnson, 2017), such that the NADPH/NADP^+^ ratio can remain stable. In other words, generated NADPH *via* PETC is immediately consumed by CBC and/or redox shuttle and recycled back to the photosystem as NADP^+^. However, photosynthesis does not occur in darkness in nature, and constant fluctuation of light intensity occurs during the day because of changes in radiation angles, cloud cover, and shade by leaf overlap in natural environments. Here, we speculate that the redox state of the NADP pool might vary in response to fluctuating light conditions and that the NADPH/NADP^+^ ratio might be at its peak because of massive NADPH generation at very high-light intensities. Similarly, we might misbelieve that NADP^+^ exclusively accumulates in the chloroplasts at night because there is no light that drives PETC to generate NADPH. However, in addition to NADPH, NADP^+^ also displays basal levels after dark acclimation for at least 30–60 min (Thormahlen et al., 2017; Hashida et al., 2018). In darkness, the redox status of NADP would be balanced by OPPP enzyme activity mediated *via* NTRC-dependent redox regulation (Michalska et al., 2009). At the timing of re-exposure to light, NADP^+^ increases before NADPH because NADP^+^ is indispensable as an electron acceptor for LEF execution. As a result, the sum of NADP^+^ and NADPH, here designated as the NADP pool, is dynamically adjusted to fluctuating light conditions under natural environments. These dynamics shed light on the importance of chloroplastic NADP homeostasis, including NADP pool size regulation, as well as redox regulation, in the research field of photosynthesis. Undoubtedly, the regulatory mechanism of chloroplastic NADP pool size depends on the balance between decreased and increased flow, rather than the redox interconversion between NADP^+^ and NADPH (Figure 2).

## Metabolic Pathways Contributing to a Decrease in NADP Pool Size

NADP metabolism, but not redox interconversion, contributes to a decrease in the NADP pool size. For example, NADP^+^ dephosphorylation results in NAD^+^ generation that can be supplied for the re-synthesis of NADP^+^ and is possibly a convenient strategy for the temporal sequestration of electron acceptors from photochemistry. Some research groups have reported NADP^+^ dephosphorylation activity in plants (Gallais et al., 2000; Hunt and Gray, 2009), but no NADP^+^ dephosphorylation activity has been shown at present in isolated chloroplasts. Deamidation converts NADP to deamide-NADP, or the so-called nicotinate adenine dinucleotide phosphate (NaADP) (Chini and Dousa, 1995). At the final step of NAD synthesis, nicotinate adenine dinucleotide (NaAD) serves in the deamidation reaction by ATP-dependent NAD synthetase (Hashida et al., 2009, 2016) so that NaADP might serve as an alternative precursor for NADP synthesis. However, NaADP molecules have never been reported in chloroplasts, and neither has NADP deamidation activity nor NaADP amidation activity been reported in chloroplasts or total leaf extracts. Nonetheless, these temporal NADP decreasing flows are attractive because they enable the re-supply of NADP for photochemistry in 1-step enzymatic reactions. On the contrary, it is known that nucleotide diphosphate linked to X hydrolase (NUDX) plays a significant role in NADP decrease because it cleaves the pyrophosphate bridge in the NAD skeleton (Yoshimura and Shigeoka, 2014). In *Arabidopsis*, AtNUDX19 aggressively participates in regulating the NADPH level as an alternative to dissipating excess reducing equivalents under intense light conditions (Maruta et al., 2016). Unlike dephosphorylation and deamidation of NADP, NUDX definitely consumes the NADP molecule, meaning that the NAD^+^ supply is indispensable for NADP^+^ re-synthesis as a photosynthetic electron acceptor in chloroplasts. Therefore, under high-light conditions, NADP^+^ has to be constitutively provided to complement and maintain the NADP pool size. The fate of NADPH consumed by NUDX under high-light conditions and decreased NADP^+^ under dark conditions has not been fully determined (Figure 2, dashed lines).

## Regulatory Factors for Increase in NADP Pool Size

Based on enzymatic property, NADP^+^ synthesis requires the substrates NAD^+^ and ATP besides NADK activity (McGuinness and Butler, 1985). If one of them is lacking, NADP^+^ supply is arrested and quiescent NADP^+^ synthesis spontaneously decreases the pool size when the NADP pool size is determined under dynamic equilibrium. Hence, the NADP increasing flow is strictly controlled by at least three factors in chloroplasts: (1) regulation of NADK activity, (2) ATP supply, and (3) NAD^+^ supply.

## Regulation of NADK Activity

Notably, light-excited electrons were mainly distributed from ferredoxin (Fd) to downstream electron acceptors in two aspects, as reducing power in metabolic reactions and as redox regulator in signal transductions. Besides the reduction of NADP^+^ by Fd-dependent NADP^+^ reductase in LEF (Figure 1, red lines), electrons are used as reducing power in the nitrate assimilation pathway and various metabolic reactions by Fd-dependent enzymes (Hanke and Mulo, 2013). In the redox signaling network, electrons could be handed over from Fd and NADPH to thioredoxin (Trx) as reducing equivalents for the downstream signaling cascade by Fd/Trx reductase and NTRC (Nikkanen and Rintamaki, 2019). Otherwise, electrons are transferred back to the plastoquinone pool by CEF (Figure 1, blue lines; Alric and Johnson, 2017). Thus, starvation of NADP^+^ as an electron acceptor results in the induction of redox regulatory flow or cyclic flow. It has recently been reported that light exposure is required for the activation of NADK2 in chloroplasts and that Fd/Trx redox signaling is directly or indirectly involved in the regulation of NADK2 activity (Hashida et al., 2018), though the regulatory mechanisms remain to be elucidated. Unlike NADK1 in cytosol and NADK3 in the peroxisome, NADK2 in chloroplasts has a long N-terminal extension, which is conserved throughout green plants (Viridiplantae, excluding glaucophytes and rhodophytes) (Li et al., 2014), but the extended amino acid sequence is originally unnecessary for enzymatic activity. However, it is still unknown whether the extension is involved in regulating NADK2 activity.

## ATP Supply

CEF operates at the maximum after dark acclimation, owing to the partial inactivation of CBC (Joliot and Johnson, 2011). As NADP^+^ shortage caused by lack of NADK2 activation results in accumulation of reduced Fd, it contributes to CEF efficiency (Breyton et al., 2006; Okegawa et al., 2008). Then, the CEF efficiency decreases shortly after light exposure (Joliot and Joliot, 2005), implying that ATP synthesis could be provided by CEF at the beginning of light perception for NADP^+^ synthesis. Therefore, CEF operation seems to be important to NADP^+^ supply and the onset of LEF, leading to light acclimation. In fact, impairment of the CEF pathway reduced the photosynthetic rate under fluctuating light conditions, causing a decrease in plant biomass (Yamori et al., 2016), possibly owing to the delay in light acclimation, namely a delay in LEF establishment. In brief, the photochemical electron transfer network is profoundly associated with NADP^+^ synthesis in chloroplasts.

## NAD^+^ Supply

NADP^+^ biosynthesis in chloroplasts is activated in the presence of sufficient ATP and NAD^+^ under light conditions. Where does NAD^+^ come from? In response to light, the chloroplastic NADP pool size doubles within 1 h by consuming an equivalent amount of NAD^+^ in chloroplasts (Hashida et al., 2018). Currently, the NAD pool size (sum of NAD^+^ and NADH) in chloroplasts is still under estimation but the sum of cellular NAD and NADP pool could increase in response to light. According to previous reports, promotion of chloroplastic NADP synthesis by *NADK2* overexpression did not significantly decrease the cellular NAD pool and in contrast, enhancement of NAD biosynthesis increased the cellular NADP pool (Takahashi et al., 2009; Hashida et al., 2010). Thus, the increase in NADP is accompanied by an increase in the total NAD and NADP pool sizes. The total pool increase indicates activation of NAD^+^ biosynthesis. This activation could be stimulated by chloroplastic NADP^+^ synthesis accompanied by NAD^+^ uptake into chloroplasts (Figure 2). As NAD biosynthesis exclusively occurs outside of chloroplasts (Hashida et al., 2010; Di Martino and Pallotta, 2011), the cytoplasmic NAD pool could decrease by the uptake. Therefore, the mechanism of NAD^+^ supply underpins the light responsive NADP increase for LEF in photosynthesis, and chloroplastic NAD^+^ transportation is a crucial pathway for light adaptation in terrestrial plants (Hashida et al., 2010, 2016; Vialet-Chabrand et al., 2017). However, chloroplasts have been less investigated with respect to NAD^+^ transport compared to mitochondria and peroxisomes (Palmieri et al., 2008; Agrimi et al., 2012). In *Arabidopsis*, NDT1 encodes a carrier protein that is capable of transporting NAD^+^ and targeting it to the inner membrane of chloroplasts (Todisco et al., 2006; Palmieri et al., 2009). At present, the physiological functions in chloroplastic NAD^+^ transportation are largely unknown. Surprisingly, a mutant lacking chloroplastic NADK activity, *nadk2*, can survive under light and conditionally grow to a size comparable to wild type, albeit having a pale green leaf (Chai et al., 2005; Takahashi et al., 2009), implying a possible complement of NADP^+^ to chloroplasts from cytosol in the *nadk2* mutant. However, neither NDT1 nor NDT2 transports NADP^+^ or NADPH according to biochemical experiments (Palmieri et al., 2009). Otherwise, NAD^+^ in chloroplasts may act as an inefficient but valuable alternative for NADP^+^ in photosynthetic electron transport. Because, *nadk2* showed a larger NAD pool size than the wild type (Takahashi et al., 2006), light-driven chloroplastic NAD^+^ transport could be promoted and the chloroplastic NAD pool might cover the increase.

## Which Pathway is Crucial for NAD^+^ Supply?

NAD^+^ also has a significant function in photosynthesis through regulation of various CBC enzymes; for example, *via* the assembly of the protein complex glyceraldehyde-3-phosphate dehydrogenase and phosphoribulokinase mediated by CP12 protein (Wedel et al., 1997). Therefore, chloroplasts contain considerable amounts of NAD^+^ and chloroplastic NAD^+^ transportation is not just for NADP^+^ synthesis. Nevertheless, the basal level of NAD^+^ cannot be accounted for by the increased NADP^+^ level in response to light, suggesting that NAD^+^ biosynthesis is also required for light acclimation (Figure 2, orange lines and captions). In the final two steps of NAD^+^ biosynthesis, nicotinate mononucleotide (NaMN) is transformed *via* adenylation to nicotinate adenine dinucleotide (NaAD), followed by amidation of NaAD to NAD^+^. However, enhancement of enzymatic activity in these steps does not particularly contribute to the NAD pool size (Hashida et al., 2010, 2016). Here, NaMN is of particular importance as an intermediate, which is a product of two independent pathways, the *de novo* and the salvage pathway. In heterologous systems, quinolinate (Qa) application with *E. coli nadC* gene encoding Qa phosphoribosyl transferase (QPT) successively increases the NAD pool size (Petriacq et al., 2012). Therefore, the supply of NaMN is a key regulator of cellular NAD pool size. In brief, the light responsive NADP increase in chloroplasts will provoke NaMN supply for NAD^+^ production. According to current knowledge, *de novo* synthesis of NaMN is executed in chloroplasts, and the generated NaMN is exported to cytosol and salvage synthesis of NaMN from nicotinate is conducted in the cytosol (Katoh et al., 2006). Therefore, the *de novo* pathway rather than the salvage pathway has better linkage to chloroplastic events (Figure 2, green and red lines).

According to a recent report, L-Asp oxidase (LASPO), which is the first enzyme of *de novo* NAD^+^ biosynthesis and converts L-Asp to imino-Asp, has intriguing biochemical properties associated with the light/dark condition (Hao et al., 2018). LASPO activity may be inhibited at night and stimulated by light exposure, as well as NADK2. Moreover, NADP^+^ can be a competitive inhibitor for LASPO, suggesting that an early step of the *de novo* NAD^+^ biosynthesis is orchestrated by chloroplastic NADP^+^ status. As the next step of *de novo* NAD^+^ biosynthesis, Qa synthase (QS) produces Qa from imino-Asp. Interestingly, QS protein harbors the cys desulfurase domain that stimulates reconstitution of the oxygen-sensitive Fe-S cluster and QS as part of the cys desulfurase complex (Murthy et al., 2007; Schippers et al., 2008). Hence, QS may monitor chloroplastic redox status in a Cys desulfurase complex and a reductive environment could stimulate QS activity. In chloroplasts, a Fe-S cluster and assembly are involved in the redox regulatory network downstream of Fd in PSI photochemistry (Van Hoewyk et al., 2007). Because the Fd/thioredoxin-*m* redox regulatory pathway negatively regulates NADP^+^ synthesis (Hashida et al., 2018), these opposite regulations may coordinate the precursor supply for *de novo* NAD^+^ biosynthesis that is required for NADP^+^ synthesis to save excess energy (ATP) consumption. Further, the above-mentioned heterologous system uses cytoplasmic ATP for NaMN synthesis. Therefore, as is the case of NADK in chloroplasts, ATP supply could be a rate-limiting factor for QPT activity. Although NaMN needs to be exported to the cytoplasm from chloroplasts for the synthesis of NAD^+^, the transport mechanism is unknown. Here, NDT1 could be a candidate NaMN transporter in addition to the ability of NAD^+^ transport because NDT1 protein can mediate NaMN as well (Palmieri et al., 2009). In brief, the light responsive NADP increase could be underpinned by the export of NaMN from chloroplasts to cytosol and the import of NAD^+^ from the cytosol to chloroplasts.

## Conclusion and Perspectives

Although genes associated with NAD^+^ and NADP^+^ biosynthesis and metabolism have been continuously characterized and identified for decades, their regulators and transport mechanisms remain largely unknown. Because NADP^+^ biosynthesis is mainly supported by the amount of available NAD^+^, NAD^+^ supply is the key regulator of NADP^+^ synthesis. Importantly, NADP^+^ synthesis proceeds on demand even though NAD^+^ synthesis is exclusively carried out in the cytosol. Therefore, inter-organelle signaling is rather important to communicate NAD^+^ demand for each location where NADP^+^ synthesis occurs. Recent studies have revealed the existence of a dynamic response of the chloroplastic NADP pool to light, underpinning NAD and NADP metabolism including degradation, metabolism, and biosynthesis. Moreover, the early steps of *de novo* NAD^+^ synthesis in chloroplasts appear to be controlled by redox regulation. Future dissection of inter-organelle NAD and NADP metabolism is thus essential for understanding the fate of chloroplastic NADP under various light conditions.

## Author Contributions

All authors listed have made a substantial, direct and intellectual contribution to the work, and approved it for publication.

### Conflict of Interest Statement

The authors declare that the research was conducted in the absence of any commercial or financial relationships that could be construed as a potential conflict of interest.
